# Temporomandibular Disorders Related to Stress and HPA-Axis Regulation

**DOI:** 10.1155/2018/7020751

**Published:** 2018-05-02

**Authors:** Kordian Staniszewski, Henning Lygre, Ersilia Bifulco, Siv Kvinnsland, Lisa Willassen, Espen Helgeland, Trond Berge, Annika Rosén

**Affiliations:** ^1^Department of Clinical Dentistry, University of Bergen, Bergen, Norway; ^2^Oral Health Center of Expertise in Western Norway, Stavanger, Rogaland, Norway; ^3^Department of Clinical Science, University of Bergen, Bergen, Norway; ^4^Department of Oral and Maxillofacial Surgery, Haukeland University Hospital, Bergen, Norway

## Abstract

Temporomandibular disorders (TMDs) are characterized by pain and dysfunction in the masticatory apparatus and the temporomandibular joint (TMJ). Previous trauma, stress symptoms, psychosocial impairment, and catastrophizing have been related to TMD. To assess if the hypothalamic-pituitary-adrenal (HPA) axis is upregulated in TMD patients, we performed a cross-sectional study with saliva from 44 TMD patients and 44 healthy sex- and age-matched controls for cortisol (*F*) and cortisone (*E*) with liquid chromatography-tandem mass spectrometry. Furthermore, we calculated the *F*/*E* ratio for the evaluation of 11*β*-hydroxysteroid dehydrogenase activity. We also assessed anxiety/depression and pain catastrophizing scores from a questionnaire that participants completed prior to the examination. We found that *F* (*P*=0.01), *E* (*P*=0.04), the *F*/*E* ratio (*P*=0.002), and the sum of glucocorticoids (*E* + *E*) in saliva (*P*=0.02) were significantly higher in the TMD group. Anxiety/depression and catastrophizing scores were also significantly higher in the TMD group (*P* < 0.0001). Our findings indicate that patients with TMDs may have an upregulated HPA axis with higher *F* secretion from the adrenal cortex. Anxiety/depression and pain catastrophizing scores were significantly higher in the TMD group, and psychological factors may contribute to chronic upregulation of the HPA axis.

## 1. Introduction

Temporomandibular disorders (TMDs) are a group of disorders associated with pain and dysfunction affecting the temporomandibular joint (TMJ) and the masticatory apparatus [1, 2]. TMDs occur predominantly in women, who are especially likely to experience more severe symptoms. TMD-associated comorbidities include fibromyalgia, irritable bowel syndrome, and depression, with trauma and stress symptoms frequently present as well [3]. Psychosocial impairment within a TMD, such as somatization and depression, is linked with pain-related disability as well as the duration of pain [4]. The Orofacial Pain Prospective Evaluation and Risk Assessment (OPPERA) study found that psychosocial factors (e.g., somatic awareness, distress, catastrophizing, pain amplification, and psychosocial stress) had a significantly higher prevalence in subjects with a TMD compared to healthy individuals [2, 5].

During the last few decades, use of physiological markers for assessing psychosocial-related disorders has increased. Stress activates the hypothalamic-pituitary-adrenal (HPA) axis, which results in a cascade of reactions leading to increased secretion of cortisol from the adrenal cortex. Research examining the HPA axis response to stress has yielded contradictory results. A meta-analysis of chronic stress and HPA-axis activity found that HPA response to stress varies with the nature and controllability of stressful stimuli as well as the individual psychiatric response [6]. The role of stress in the etiology and persistence of TMD remains unclear. However, dysregulation of the HPA axis has been correlated with TMD in several studies [7–9]. Accordingly, analysis of cortisol (*F*) levels in saliva may provide a means for examining HPA-axis activity.

Salivary *F* levels follow circadian fluctuations, and these variations can be used to create a curve depicting unbound free and total cortisol in serum [10]. However, previous analyses of *F* in saliva from TMD patients have given variable results. Some researchers have found elevated *F* values in association with TMD [11, 12], while others have not found any significant difference in comparison to a control group [13]. Analyses using immunoassay methods [11–15] have also been undertaken to measure *F* in saliva from subjects with a TMD. These methods do not separate cortisol (*F*) and cortisone (*E*), which have structural similarities but unequal biological activities. Recent *F* and *E* analyses based on liquid chromatography-tandem mass spectrometry (LC-MS/MS) are now available [16].

The primary objective of this study was to assess the stress levels in TMD patients based on an upregulated HPA axis and compare the results with healthy individuals. Secondary objectives were to analyze the saliva for *F* and *E* and the scores for self-reported anxiety/depression and catastrophizing from a questionnaire. The hypothesis was that TMD patients have an upregulated HPA axis shown by increased psychological scores and increased level of cortisol in saliva.

## 2. Materials and Methods

### 2.1. Study Design

The present study is a clinical cross-sectional study, which was a part of a multidisciplinary investigation of TMD patients at Haukeland University Hospital, sponsored by the Norwegian Ministry of Health [17]. Ethical approval was granted by the Regional Ethical Review Board South East (2015/930), in accordance with the Helsinki Declaration (1964). A written informed consent was received from all subjects.

### 2.2. Participants

All TMD patients (*n*=60) were referred by their general practitioner to the National TMD project in Bergen, Norway. The subjects were from all regions in Norway and were consecutively included in the project during the years of 2013–2015. Patients were included, examined, and evaluated based on the severity and duration of symptoms, both for pain and dysfunction and for consequences. Six specialists representing several disciplines, who created an individual treatment proposal for each patient, performed the examination. The investigation included pain intensity and duration, functional impairment (general and jaw-specific), effect on quality of life, and presence of extended periods of sick leave. Inclusion criteria were long-term TMD-related pain. Furthermore, inclusion was based on the examination; thus, patients with and without functional impairment were included. Exclusion criteria were non–TMD-related orofacial pain, relevant drug dependence problems, and obvious psychiatric diagnoses.

A healthy sex- and age-matched control group (*n*=60) was recruited for comparison with the TMD patients, during 2016. A majority of the control group consisted of employees and students from the Department of Clinical Dentistry at the University of Bergen, who were not affiliated with the study research group. The remaining members of the control group were recruited from the general population in Bergen, Norway. The subjects gave their informed consent to participate in the study. Inclusion criteria for the control group was age 20 years or older and age- and sex-matched with the TMD patient group. Exclusion criteria were TMD symptoms or other musculoskeletal pain and symptoms in the head and neck area. Individuals in the control group were anonymized.

### 2.3. Questionnaire

TMD patients completed a comprehensive questionnaire prior to clinical examination. The questionnaire covered medical history, socioeconomic history, and lifestyle factors and included tools to assess psychosocial factors, specifically the Hospital Anxiety and Depression Scale (HADS) [18] and a 2-item version of the Coping Strategies Questionnaire [19] regarding catastrophizing. The healthy individuals completed a shortened version of the same questionnaire.

### 2.4. Saliva Samples and Analyses

Saliva samples were collected in the morning with the Salivette Cortisol Code Blue test kit (Sarstedt Darmstadt, Germany) and stored at −80°C until analysis. *F* and *E* were determined by liquid chromatography-tandem mass spectrometry (LC-MS/MS) at the Core Facility for Metabolomics, University of Bergen. Sample processing was completely robotized (Hamilton Robotics, Inc., Reno, NV, USA). Briefly, 20 *μ*L of internal standard (Cortisol-2,3,4-^13^C_3_) was added to 100 *μ*L of human saliva, which was subjected to liquid-liquid extraction with 480 *µ*L of ethylacetate-heptane (80 : 20, v/v). The supernatant (380 *µ*L) was subsequently washed with 50 *µ*L of sodium hydroxide (0.1 M). Next, 280 *µ*L of supernatant was removed and evaporated to dryness under nitrogen flow and then reconstituted in 100 *µ*L of a 0.01% aqueous solution of formic acid : methanol (50 : 50, v/v). Samples were then analyzed on a Waters ACQUITY UPLC system connected to a Waters Xevo TQ-S tandem mass spectrometer (Waters, Milford, MA, USA). The compounds were separated on a C-18 BEH phenyl column from Waters (100 × 2.1 mm column, 1.7 mm particle size), which was developed by gradient elution over 5.5 min, using an aqueous solution of formic acid and acetonitrile as mobile phases. Formic acid adducts were detected in negative multiple reaction-monitoring mode. A potential source of bias is that the TMD patients likely experienced more stress prior to the examination compared to the controls because the majority of the controls were examined at their ordinary workplace.

### 2.5. Statistical Analyses

All statistical analyses were performed in STATA. Mean, median, range, and standard deviation (SD) for all variables in both groups were calculated. A paired *t*-test was used to calculate the *P* value of no difference in *F*, *E*, *F*/*E* ratio, and *F* + *E* between the TMD group and the control group. A Wilcoxon signed rank test was used to calculate the *P* value of no difference in HADS and catastrophizing scores between the TMD group and the control group. A linear multiregression between *F* and psychosocial factors in both groups was performed as well as a linear correlation (*R*) with associated *P* values between GC levels and psychosocial factors.

## 3. Results

### 3.1. Demographic Data

**Figure 1:**
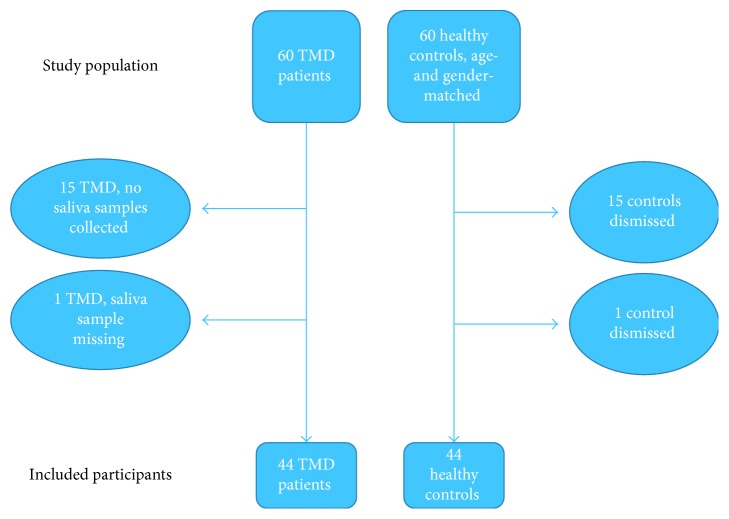
Flow chart of the study population: TMD patients and healthy controls.

The multidisciplinary investigation [17] consisted of 60 patients, all experiencing severe TMD symptoms, and 60 healthy control subjects. Because no saliva sampling was done for the first 15 TMD patients and one saliva sample was missing from the patient group, the population in the present study ended up with 44 TMD patients and 44 healthy controls (Figure 1). The patients were aged 20–69 years, with a mean age of 44 years. The control subjects were aged 23–71 years with a mean age of 46 years. Both groups consisted of 38 women and 6 men.

### 3.2. Saliva Samples and Analyses

The TMD patient group had a mean saliva-sampling time point of 2 h, 52 min after awakening. The saliva samples were mostly collected at 9:00 AM but a few were collected at 11:00 AM owing to logistic factors. All subjects in the control group collected saliva 2 h, 45 min after awakening, matching the mean sampling time of the TMD patient group. Saliva samples from the control group were collected between 8:00 AM and 10:00 AM.

The transitions monitored under LC-MS/MS analyses were 405.22→329.24 for *E* and 407.24→331.26 for *F*. The linearity range was 0.7–100 nmol/L for *E* and 0.3–50 nmol/L for *F*. Accuracy was between 87% and 110%, and total imprecision was <10%.

### 3.3. Stress Scores and Glucocorticoids in Saliva

Our most important finding was that *F* in saliva was significantly higher in the TMD group compared to the control group (*P*=0.01) (Table 1). *E* (*P*=0.04), the *F*/*E* ratio (*P*=0.002), and the sum of GC (*F* + *E*) in saliva (*P*=0.02) were also significantly higher in the TMD group. Stress scores from questionnaires were significantly higher in the TMD group, including pain catastrophizing (*P* < 0.0001) and HADS (*P* < 0.0001) (Table 2). Pain catastrophizing score in the TMD group was negatively correlated with *E* and *F* + *E* (*P*=0.033 and *P*=0.047, resp.); however, no association between *F* and pain catastrophizing was found (Table 3). In the control group, we observed a significant correlation between depression score and *F* + *E* (*P*=0.045). No other associations between the GC levels in saliva and psychosocial factors were found in the control group (Table 4).

## 4. Discussion

In this study, we found that *F* and *E* levels in saliva are significantly higher in TMD patients compared to healthy individuals. Our results were obtained by LC-MS/MS analysis. Compared with immunoassays, LC-MS/MS has much higher specificity and thus permits identification and quantification of *F* and *E* [16, 20, 21]. To our knowledge, this study is the first to determine *F* in TMD by LC-MS/MS and the first to investigate the sum and ratios of different GCs in TMD patients. However, the LC-MS/MS indicates significantly lower *F* levels than immunoassays due to a lower incidence of cross-reactions [22]. The correlation between LC-MS/MS and immunoassays is poor [16], and the *F* and *E* levels measured in this study are consequently not directly comparable to those from previous studies of TMD patients using immunoassays. Accordingly, our study may also contribute to the general assessment of salivary levels of *F* and *E* in healthy and diseased subjects.

*F* levels in healthy individuals follow circadian fluctuations. The lowest value occurs during early sleep and levels rise until awakening and then rise even faster in the cortisol awakening response. The peak value occurs approximately 30–45 min after awakening [23, 24]. Our saliva samples had a mean sampling time 2 h, 52 min after awakening in the TMD group and 2 h, 45 min in the control group. Accordingly, *F* levels from our patients and controls were not directly comparable to previous TMD studies because of the diurnal decrease in *F* levels after peaking in addition to lower *F* levels being expected from LC-MS/MS compared with immunoassays.

Many studies have reported elevated *F* levels in TMD patients compared to healthy individuals. A significantly higher daytime *F* value in plasma was reported in subjects with TMD compared to healthy controls [14]. Analysis of saliva from TMD patients also revealed elevated *F* levels [11, 12]. Significant higher *F* levels as a response to experimental stress in subjects with TMD has also been reported [15]. In contrast, some researchers have not found significant differences in salivary *F* levels related to TMD [13]. In a study examining hair *F* concentration, even lower values of *F* were found in subjects with TMD [7].

Elevated or lowered basal *F* levels may reflect changes in the regulation of the HPA axis, which is discussed in other TMD studies and in several studies of stress-related and chronic pain disorders [7, 9, 14, 15, 25–32]. A significantly higher rise in salivary *F* in response to experimental stress has been reported in a TMD group compared to a healthy control group [15]. An opposite finding within a subgroup separate from the TMD group in the same study showed slightly lower, but nonsignificant, salivary *F* levels compared to the control group at all measuring points. No significant differences in basal *F* levels existed between the TMD and control groups before the stress exposure [15]. However, no difference in salivary *F* levels was reported as a response to experimental pain in a TMD group compared to a control group. Nevertheless, an association between high pain-catastrophizing scores and high *F* response to pain was observed although basal morning *F* was lower in association with high pain catastrophizing in both TMD and controls [25]. In our study, we showed that not only *F*, but also *E* and the sum of both GCs (*F* + *E*), was significantly higher in the TMD group. This finding means that the total sum of GCs is higher in the TMD group and supports the theory of an upregulated HPA axis, with higher *F* secretion from adrenal cortex. The high level of the inactive hormone *E* may be the result of enzymatic conversion of *F* by 11*β*-hydroxysteroid dehydrogenase type 1 (11*β*-HSD-1) in the glandula parotis.

Another possible explanation of higher *F* levels in TMD patients may arise from suppressed negative feedback of the HPA axis, as seen in major depression [27]. An exaggerated *F* response to CRH as well as higher basal *F* levels has been reported for patients with irritable bowel syndrome [28]. Since we did not perform any suppression tests in our study, we could not evaluate the negative feedback of the HPA axis for comparison.

The *F*/*E* ratio is an indicator of 11*β*-HSD activity, which has previously been measured in early morning saliva sample and found to be 0.24 [33], 0.15 [34], and 0.20 [35]. The active molecule *F* is converted to an inactive form *E* in parotid tissue by the enzyme 11*β*-HSD-1 and a reverse conversion by 11*β*-HSD-2. Our calculations resulted in a *F*/*E* ratio of 0.26 in TMD patients compared to 0.2 in controls. The difference may be explained by decreased activity of 11*β*-HSD-2 in TMD patients or 11*β*-HSD-2 saturation at a high substrate concentration [35]. Enzyme saturation has previously been indicated by scatter plots with curve fitting [33, 35], showing that the increase in salivary *E* is nonlinear with the increase of salivary *F* at high *F* concentrations. For example, an elevated *F*/*E* ratio was reported in a study of apparent mineralocorticoid excess [36], and *F*/*E* ratios in urine were reported to be significantly higher in depressed patients compared to healthy individuals [37]. In fetoplacental tissue, 11*β*-HSD-2 has a key function in neurobehavioral development, and loss of its function has resulted in lifelong anxiety in mice [38]. Given that 11*β*-HSD-2 is supposed to protect the mineralocorticoid receptor from GC binding [39], examining blood pressure in TMD patients in future studies could be interesting.

Psychosocial factors such as stress, anxiety, and depression may influence the HPA axis as well, although the response seems unclear and inconsistent. Stress may potentially be an important factor in the etiology of TMD [11]. The prevalence of physical and psychological stressors in TMD is high, and they may contribute to dysregulation of the HPA axis [8]. However, no significant differences in salivary morning *F* were reported from a study of 30 young women with TMD, although the TMD subjects appeared more psychologically distressed compared to healthy individuals [13]. Subjects with TMD also had a significantly higher stress score, despite apparently lower *F* levels, which were measured through hair analysis [7]. However, *F* levels in hair may reflect stress and *F* output over time, while salivary *F* reflects the same variables at the point of measurement. The TMD patients in our study scored significantly higher on HADS and pain-catastrophizing questionnaires, which could reflect higher stress levels that potentially contribute to an upregulation of the HPA axis. Still, we did not find any significant correlation between anxiety, depression, or catastrophizing scores and *F* levels. This outcome may be due to the presence of many other factors influencing *F* levels. Nevertheless, we found a significantly negative association between pain-catastrophizing score and both *E* and the sum of GCs (*F* + *E*). *F* was also lower with higher pain catastrophizing in the TMD group, but the association was nonsignificant. Nevertheless, the findings from our study are comparable with a previous study in which lower basal *F* was associated with high pain catastrophizing [25]. Nonsignificantly higher catastrophizing scores in a subgroup of TMD patients with low *F* levels have also been reported [15]. However, we did not see lower *F* levels correlated to anxiety or depression in the TMD group. In the control group, we observed a significant correlation between depression score and *F* + *E*, though the majority in the control group had a depression score that ranged zero to very low, and the association has probably low scientific value. We could not find any other correlations between GC levels and any psychological factor in the control group. A recent review on stress in chronic pain patients highlighted that several types of HPA-axis dysregulation can occur in chronic stress and pain conditions, leading to a HPA-axis stress response that cannot be determined by basal *F* levels only [40].

The role of stress in the etiology of TMD remains unclear. The effect of stress in TMD patients may result in a complex and multifactorial response by biological systems, including neuroendocrine function and psychosocial and physical adjustments [9].

## 5. Conclusion

In summary, we report that a group of TMD patients had significantly higher *F* and *E* levels compared to a healthy control group. This finding may indicate that TMD patients have an upregulated HPA axis. Anxiety/depression and pain-catastrophizing scores were significantly higher in the TMD group, and they may potentially indicate chronic upregulation of the HPA axis. Based on these results, the hypothesis that TMD patients have an upregulated HPA axis may be approved. More research is needed to confirm the activity of the HPA axis in TMD patients. In future studies, it would be interesting to collect samples at several time points to compare their diurnal *F* rhythm. Examination of the *F* response to experimental stress would be expedient, as would suppression by dexamethasone and further investigation of 11*β*-HSD; blood pressure would be of great interest.

## Figures and Tables

**Table 1:** Glucocorticoid levels in saliva of TMD patients and healthy controls, analyzed with liquid chromatography-tandem mass spectrometry (LC-MS/MS). A paired *t*-test resulted in significant higher levels of cortisone (*E*) and cortisol (*F*), as well as the ratio of *F*/*E* and the sum of *F* + *E*, in TMD patients.

Glucocorticoids	Cortisone (*E*) (nmol/L)	Cortisol (*F*) (nmol/L)	*F*/*E* (ratio)	*F* + *E* (nmol/L)
TMD (*n*=44)				
Mean	26.31	7.17	0.26	33.48
Median	24.83	6.29	0.26	31.37
Range	13.17–47.05	2.24–27.04	0.14–0.66	15.41–67.77
SD	8.61	4.56	0.09	12.49
Control (*n*=44)				
Mean	22.91	4.90	0.20	27.81
Median	21.56	3.81	0.18	25.35
Range	10.54–74.38	1.42–28.21	0.10–0.53	15.68–102.59
SD	9.74	4.37	0.09	13.91
*P* value (paired *t*-test)	0.041	0.01	0.002	0.02

**Table 2:** Results from the questionnaires Hospital Anxiety and Depression Scale (HADS) and Coping Strategies Questionnaire regarding catastrophizing, assessed in the TMD patients and controls. A signed rank test resulted in significant higher score on all parameters in the TMD patient group.

Psychosocial scores	Mean	Median	Range	SD	*P* value (signed rank)
Catastrophizing (0–12)					<0.0001
TMD	7.88	8.0	1–12	2.95	
Control	1.39	0.0	0–11	2.64	
Anxiety (*A*) (0–21)					0.0002
TMD	7.73	7.0	0–20	5.11	
Control	3.35	2.0	0–12	3.22	
Depression (*D*) (0–21)					<0.0001
TMD	6.28	5.0	0–19	5.07	
Control	1.70	1.0	0–9	2.32	
*A* + *D* (HADS) (0–42)					<0.0001
TMD	14.25	13.0	0–39	9.76	
Control	5.05	3.5	0–19	4.85	

**Table 3:** Linear correlation (*R*) with associated *P* values between glucocorticoid levels and psychosocial factors in the TMD group. Pain-catastrophizing score was significant, negatively correlated with *E* and the sum of glucocorticoids (*F* + *E*) (*P*=0.033 and *P*=0.047, resp.). No significant association between *F* and pain catastrophizing was found, neither any significant associations between the other parameters of glucocorticoid levels in saliva and psychosocial factors.

TMD group	Cortisone (*E*)	Cortisol (*F*)	*F*/*E*-ratio	*F* + *E*
Catastrophizing score				
*R*	−0.323	−0.230	−0.080	−0.305
*P* value	0.033	0.138	0.611	0.047
Anxiety (*A*) score				
*R*	−0.089	0.125	0.247	−0.016
*P* value	0.566	0.420	0.107	0.919
Depression (*D*) score				
*R*	−0.091	0.036	0.128	−0.049
*P* value	0.563	0.821	0.415	0.753
*A* + *D* (HADS) score				
*R*	−0.042	0.123	0.211	0.016
*P* value	0.785	0.426	0.169	0.919

**Table 4:** Linear correlation (*R*) with associated *P* values between glucocorticoid levels and psychosocial factors in the control group. Depression score was significantly associated with the sum of glucocorticoids (*F* + *E*) (*P*=0.045). No significant associations between the other parameters of glucocorticoid levels in saliva and psychosocial factors were observed.

Control group	Cortisone (*E*)	Cortisol (*F*)	*F*/*E*-ratio	*F*+*E*
Catastrophizing score				
*R*	0.111	0.147	0.175	0.124
*P* value	0.473	0.340	0.256	0.422
Anxiety (A) score				
*R*	0.187	0.171	0.044	0.185
*P* value	0.225	0.266	0.778	0.231
Depression (*D*) score				
*R*	0.313	0.269	0.010	0.304
*P* value	0.039	0.077	0.519	0.045
*A* + *D* (HADS) score				
*R*	0.273	0.242	0.077	0.268
*P* value	0.073	0.113	0.620	0.079

## Data Availability

The data used to support the findings of this study are available from the corresponding author upon request.
